# Clinical Evaluation of an Affordable Handheld Wavefront Autorefractor in an Adult Population in a Low-Resource Setting in the Amazonas

**DOI:** 10.3390/vision9040094

**Published:** 2025-11-06

**Authors:** David Tayah, Ricardo Noguera Louzada, Pedro Lucas Machado Magalhães, Youssef Tayah, Dillan Cunha Amaral, Chow Wang Ming Shato, Daniel Oliveira Dantas, Milton Ruiz Alves

**Affiliations:** 1 Department of Ophthalmology, Faculty of Medicine, Federal University of Amazonas, Manaus 69080-900, AM, Brazil; chow@usp.br; 2 Department of Ophthalmology and Otorhinolaryngology, School of Medicine, University of São Paulo, São Paulo 05508-220, SP, Brazil; milton.alves@hc.fm.usp.br; 3 Department of Ophthalmology and Otorhinolaryngology, Faculty of Medicine, Federal University of Rio de Janeiro, Rio de Janeiro 21941-853, RJ, Brazil; dillanamaral@ufrj.br; 4 Faculty of Medicine, Institute of Medical Education, Angra dos Reis 23914-360, RJ, Brazil; pedrolucasm@mail.tau.ac.il; 5 School of Medicine, Universidade Nilton Lins, Manaus 69058-030, AM, Brazil; youssef_sabba@hotmail.com; 6 Department of Computer Science, Federal University of Sergipe, São Cristóvão 49100-000, SE, Brazil; ddantas@dcomp.ufs.br

**Keywords:** ophthalmologic diagnostic techniques, ocular refraction, comparative study, eye health

## Abstract

This study evaluates the ability of the QuickSee Free (QSF) portable autorefractor (PlenOptika) to detect and measure refractive error compared to subjective clinical refractometry (SCR) in a Brazilian adult population in a low-resource setting in Amazonas. A total of 100 participants aged 18–65 years underwent visual acuity screening and autorefraction with and without cycloplegia using the QSF, alongside a complete ophthalmic examination including SCR. Refractive error measurements included spherical component (SC), cylindrical component (CC), cylindrical axis (CA), spherical equivalent (SE), and vector powers (MV90 and MV135). Accuracy was assessed for hyperopia ≥ +2.00 D, myopia ≤ −0.75 D, astigmatism ≥ 1.00 DC, and anisometropia ≥ 1.00 D using receiver operating characteristic (ROC) curve analysis. The area under the curve for detecting significant refractive errors ranged from 0.538 to 0.930. The mean difference between QSF without cycloplegia and SCR was −1.08 ± 1.17 D for SC and −1.15 ± 1.15 D for SE (*p* < 0.0001), and with cycloplegia, it was −0.81 ± 1.07 D and −0.83 ± 1.02 D, respectively. The QSF exhibited a moderate negative bias for both SC and SE with and without cycloplegia, underestimating these values, but it showed good predictability for detecting refractive errors in a low-resource setting.

## 1. Introduction

Uncorrected refractive errors (UREs) cause more than half of the visual impairments (VIs) in persons globally and are the second most common cause of blindness [1,2]. UREs are a heavy burden on affected individuals and reduce educational opportunities, productivity, and quality of life for millions of people [3,4,5]. In a random Brazilian population, 13.8% had uncorrected refractive errors, of which there was reversal of the VI condition in about 50% of them with a simple optical prescription [6]. URE is one of the most easily treatable causes of VIs [7,8].

In Brazil, the public unified health system has not yet been structured to provide ophthalmological care for URE patients. There is a lack of human resources capable of implementing actions to promote eye health, as well as a shortage of physical infrastructure and equipment for refraction exams [9,10,11]. The Brazilian Council of Ophthalmology points to the need to carry out new actions to control the growing flow of demand and expand the access of URE patients to ophthalmology services [9,12].

The lack of refractive exams and the non-availability of corrective lenses remain major barriers to the non-correction of ametropias [13,14]. In this scenario, portable autorefractors represent a promising alternative to overcome the barriers related to infrastructure, cost, and access to specialized professionals. Their portability and ease of use enable deployment in underserved and remote areas, supporting large-scale screening programs and the timely delivery of spectacles [15,16]. By enabling the detection of URE outside conventional clinical environments, such platforms can bridge existing care gaps and provide practical solutions for low- and middle-income countries [17].

Thus, this study aims to evaluate the ability of the QuickSee Free (QSF) (PlenOptika), a portable autorefractor based on Shack–Hartmann wavefront technology to detect and measure refractive error relative to gold-standard subjective clinical refractometry (SCR) in individuals aged between 18 and 65 years in a low-resource setting in Manacapuru, a city in the interior of Amazonas, Brazil.

## 2. Materials and Methods

This research was approved by the Ethics Committee of the University Nilton Lins of Amazonas, Manaus, Brazil (CAAE:78664624.40000.5015 with file number 6.751.904), and followed the tenets of the Declaration of Helsinki. Informed consent was obtained from the participants or their parents or legal guardians.

A total of one hundred individuals aged 18 to 65 years from the municipality of Manacapuru, Amazonas, Brazil, were selected for the study. Information was collected regarding age, gender, and ophthalmological findings. Subjects with best-corrected visual acuity (VA) < 20/20 in either eye, regardless of their refractive status, or with any ocular disease, were excluded from the study. The ophthalmological measurements were obtained by a trained nurse in the following sequence for both groups: (i) VA (Snellen chart at 6 m) without optical correction, (ii) three QSF refraction measurements without cycloplegia, (iii) three QSF refraction measurements with cycloplegia, (iv) SCR under cycloplegia with a Greens refractor, (v) VA with correction, (vi) slit lamp biomicroscopy, and (vii) fundoscopy. Cycloplegia was achieved by instilling a drop of 1% cyclopentolate (Cyclopentolate 10 mg/mL, Allergan–AbbVie, Inc., North Chicago, IL, USA), which was complemented after 5 min with a drop of 1% tropicamide (Mydriacyl 1%, Novartis, Basel, Switzerland). A refraction examination was performed 30 min after the first instillation.

The QSF used in this study measures refractive errors in eyes with pupils from 2 to 8 mm; the exam acquisition time is 5 to 10 s; the spherical range was from −13.00 D to +10.00 D in increments of 0.01 D, 0.125 D, and 0.25 D; the cylindrical range was from −8.00 DC to +8.00 DC with increments of 0.01 DC, 0.125 DC, and 0.25 DC; and the axial range was 0–180° with increments of 1°, 5°, and 10°. It is already factory-calibrated and does not require re-calibration in the field. It stores more than ten thousand exams. It weighs 650 g and has the following dimensions: 30 cm × 5.5 cm × 18 cm. QSF is registered as an FDA Class I 510 (k) exempt medical device.

The refraction measures were averaged and compared. Data were collected exclusively from the right eye (RE) to prevent issues related to the interdependence of observations from both eyes of an individual. For the refractive error analysis, the spherical component (SC) was expressed in spherical diopters, the cylindrical component (CC) in cylindrical diopters, and the main axis of the cylinder (CA) in degrees. To calculate average values and perform statistical analyses, readings were converted into a spherical equivalent (SE), which corresponded to the spherical value plus half the astigmatism value. In addition, the SC and CC components were converted into power vectors according to the Naeser equation: MV90 = m (sen^2^α − cos^2^α), where MV90 is the magnitude vector on the 90° axis, m is astigmatism in diopters, and α is the meridian of astigmatism in degrees (vertical and horizontal refraction components) [18]. The equation MV135 = m (sen^2^ (α − 45°) − cos^2^ (α − 45°) allows for the calculation of the difference between diopter components projected on the 135° axis and the 45° axis. To maintain the SE format, the MV components were divided in half.

A previous study reported that the SD of the SE obtained with QSF minus subjective refraction, both with cycloplegia, is 0.60. Using this standard deviation value, a power analysis indicated that a sample size of 92 individuals would be sufficient to detect a difference of 0.25 D between the two groups with 0.8 power and a significance level of 0.05 (Supplementary Figure S1). The data were tested for normality using the Shapiro–Wilk test. In every pair to be compared, at least one returned a *p*-value smaller than the significance level of 0.05, indicating that the distributions are not normal. So, the comparisons (between measurements) were performed using Wilcoxon’s signed-rank test.

The statistical analysis was performed using the R statistical computing environment, version 4.4.1. The difference in SE values was calculated as the QSF SE value minus the SCR SE value. The same procedure was applied to MV90 and MV135, respectively. Comparisons between measurements were performed using Wilcoxon’s signed-rank test. The univariate analysis, which compared the difference between pairs of exams, was performed using Wilcoxon’s nonparametric test. Bivariate and trivariate analyses were performed using Hotelling’s test.

The accuracy of the refraction measures by QSF, without and under cycloplegia, was evaluated when identifying significant refractive errors (hyperopia ≥ +2.00 D, myopia ≤ −0.75 D, astigmatism ≥ 1.00 DC, and anisometropia ≥ 1.00 D), using ROC curve analysis with the SCR as the gold standard, with data obtained from both eyes. An ROC curve plots sensitivity against the false-positive rate (i.e., 1-specificity), where each point represents values obtained at a different cutoff value from a continuous or ordinal measure. We used multivariate logistic regression techniques to perform the ROC analysis. The level of statistical significance was set at 5% (*p* < 0.05). The Bland–Altman analysis comparing the SE of QSF and subjective refraction indicated a bias of −0.835 with a confidence interval between −1.0382 and −0.6318 with a *p*-value < 0.0001. This means that QSF tends to underestimate the spherical equivalent in 0.835 D (Supplementary Figure S2).

## 3. Results

The mean age of the sample group was 37.5 years, the standard deviation of age was 12.90 years, and the median age was 37 years. Regarding gender, 43 adults were male (43%) and 73 were female (73%). The mean ± SD of the SE of the refractive error was −0.91 ± 1.69 D, ranging from −6.00 D to +5.50 D. Statistical comparisons were made between the refractometric measurements obtained by QSF, without and under cycloplegia, with those obtained by SCR. The results are shown in Table 1, Table 2, Table 3 and Table 4.

**Figure 1:**
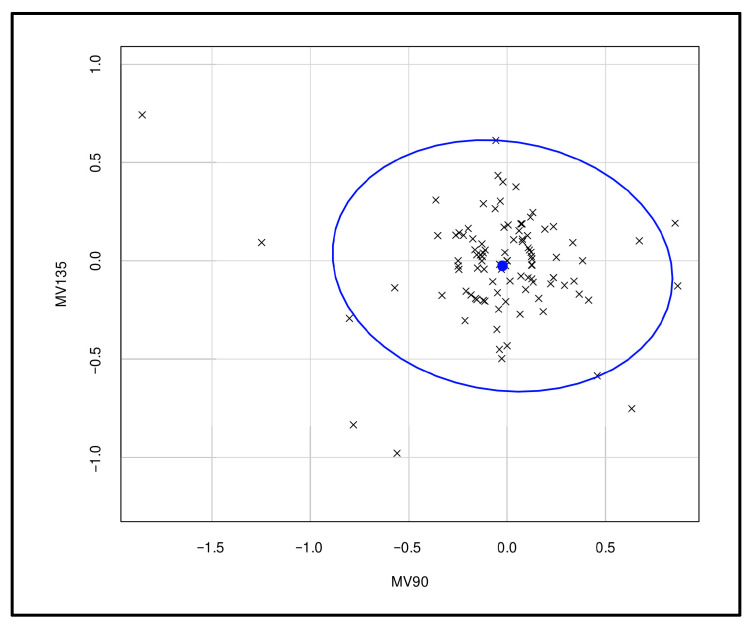
Bivariate analysis of the influence of the parameters’ magnitude vector 90 and magnitude vector 135 on differences between right-eye refraction values obtained by QSF under cycloplegia and SCR in 100 healthy adults. Manacapuru, Amazonas, 2025. MV90: magnitude vector on 90° axis; MV135: difference between diopter components projected on the 135° axis and the 45° axis. The ellipsis indicates the region where 95% of the data is expected to fall when following a bivariate normal distribution.

A bivariate analysis was performed to evaluate the influence of astigmatism on the differences between the refraction values obtained by QSF under cycloplegia and SCR (Figure 1).

**Figure 2:**
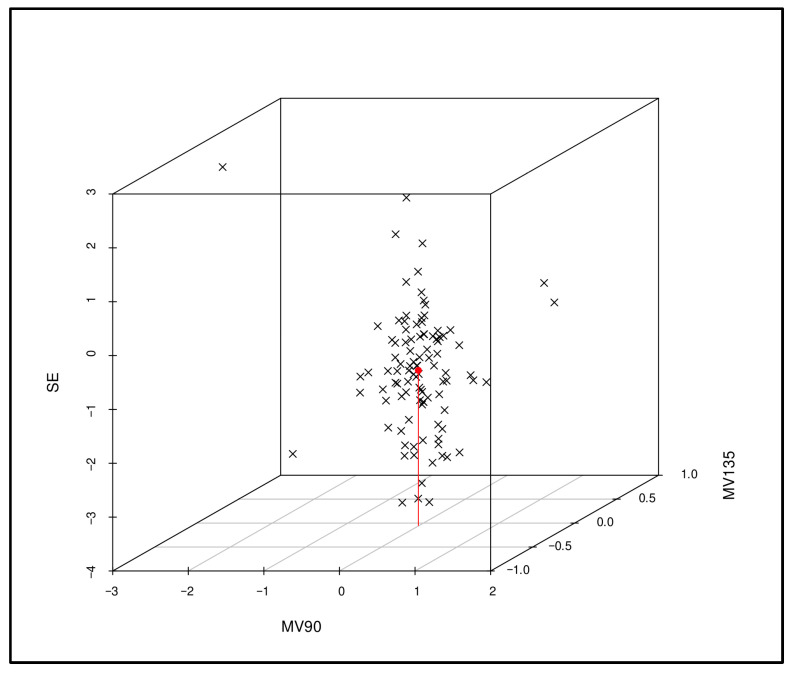
A trivariate analysis and a 3D plot assessed the relationship between SE, MV90, and MV135 and their influence on differences between right-eye refraction values obtained using QSF under cycloplegia and SCR. Manacapuru, Amazonas, 2025. SE: spherical equivalent; MV90: magnitude vector on the 90° axis, magnitude vector 135: difference between diopter components projected on the 135° axis and the 45° axis. The superior extremity of the red line indicates the average of the 3D points.

In the trivariate analysis, a 3D plot was used to assess the relationship between the parameters SE, MV90, and MV135 and their influence on differences between the right-eye refraction values obtained using QSF under cycloplegia and SCR (Figure 2).

The conversion of refraction in vector values for the conventional form revealed that, on average, the difference between the QSF measurement under cycloplegia and the SCR was −0.80 DE with −0.069 × 66.61°, with SE of −0.83 D for each right eye.

**Figure 3:**
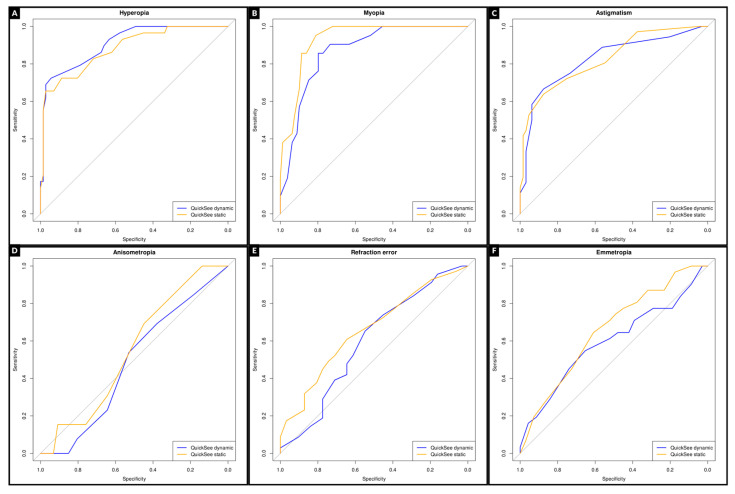
Receiver operating characteristic (ROC) curves for the QuickSee (QSF), with and without cycloplegia, in detecting significant refractive errors using subjective cycloplegic refraction (SCR) as the gold standard: (**A**) hyperopia (≥+2.00 D), (**B**) myopia (≤−0.75 D), (**C**) astigmatism (≥1.00 DC), (**D**) anisometropia (≥1.00 D), (**E**) refractive error, and (**F**) emmetropia. All plots were generated using data from the same sample of 100 adults aged 18 to 65 years. The diagonal line indicates the performance of a random classifier.

We assessed the accuracy of QSF, without and under cycloplegia, for detecting significant refractive errors (hyperopia ≥ +2.00 D, myopia ≤ −0.75 D, astigmatism ≥ 1.00 DC and anisometropia ≥ 1.00 D), using ROC curve analysis with the SCR as the gold standard (Figure 3). The values of AUC without and under cycloplegia were for hyperopia (0.882 and 0.909), myopia (0.869 and 0.930), astigmatism (0.825 and 0.826), and anisometropia (0.538 and 0.550). ROC curve analysis showed the following optimal cutoffs: static QuickSe hyperopia +1.125 D, myopia −1.125 D, astigmatism −1.125 D, and anisometropia 0.375 D; dynamic QuickSee, hyperopia +0.500 D, myopia −1.875 D, astigmatism −1.125 D, and anisometropia 0.375 D. The lower myopia cutoff in the dynamic mode is consistent with its greater negative bias in SE compared to subjective refraction (−1.15 D vs. −0.83 D).

## 4. Discussion

URE causes more than half of the VIs in persons globally and is the second most common cause of blindness [2]. Global estimates by Hashemi et al. reported a prevalence of myopia of 11.7% in children and 26.5% in adults, hyperopia of 4.6% in children and 30.9% in adults, and astigmatism affecting 14.9% of children and 40.4% of adults worldwide, underscoring the substantial public health burden of uncorrected ametropias [19]. It is estimated that, globally, only 36% of people with VIs due to URE have received access to an appropriate intervention [20]. Implementing programs to eliminate this important cause of VIs is urgent [21]. The WHO global initiative “VISION 2020: The right for sight” included refractive errors as one of five priorities to eliminate preventable blindness worldwide [22]. Therefore, there is a need to break down all barriers to access the identification and optical correction of significant refractive errors [9,11,12].

This study compared the refraction measurements, both with and without cycloplegia, of QSF and SCR in individuals aged 18 to 65 years. The univariate analysis of the differences between the values of RE refraction measured without cycloplegia by the QSF and SCR showed that the mean ± SD of the differences in SC and SE were very high, at −1.08 ± 1.17 D (*p* < 0.0001) and −1.15 ± 0.44 D (*p* < 0.0001), respectively, indicating differences with clinical relevance. The differences in vector magnitude at the 90° and 135° meridians were less than 0.25 D, deemed clinically irrelevant (Table 3 and Table 4). Nevertheless, the results of the univariate analysis of the differences between the RE refraction values measured by QSF under cycloplegia and SCR showed that the mean ± SD of the differences in SC and SE was −0.81 ± 1.07 D (*p* < 0.0001) and −0.83 ± 1.02 D (*p* < 0.0001). It should be noted that differences lower than 0.50 D are of no clinical relevance [23]. The differences in vector magnitude at the 90° and 135° meridians were also less than 0.25 D, deemed clinically irrelevant (Table 3 and Table 4). Rubio et al. compared the results of the refraction performed without cycloplegia for an affordable handheld wavefront autorefractor with those of SCR conducted on 54 patients (33.9 ± 14.1 years of age) with SE ranging from −7.25 to 4.25 D (mean ± SD, −0.93 ± 1.95 D) [24]. The mean differences between the portable autorefractor and SCR were 0.09 ± 0.39 D for M, −0.06 ± 0.13 D for J0, and 0.02 ± 0.12 D for J45. The device agreed within 0.50 D of subjective clinical refraction in 87% of the eyes for SE power. In our study, the mean differences in the refractive values of the QSF without cycloplegia were −0.13 ± 0.83 for CC, 0.00 ± 0.44 for MV90, and −0.02 ± 0.21 for MV135; however, the difference for SE power was very high −1.15 ± 1.15 D.

Although there are no articles measuring refraction with this new system, several studies using similar devices have reported comparable findings and provided a useful context for interpretation. For example, comparisons of wavefront aberrometers and autorefractors with subjective refraction in adults have demonstrated good agreement, although discrepancies of ±0.50 D or greater were noted in a small percentage of cases [25]. In pediatric populations, handheld instruments such as the Spot Vision Screener showed excellent correlation with cycloplegic retinoscopy for spherical equivalent but lower accuracy for cylinder values [26]. Likewise, the binocular wavefront optometer has been shown to deliver both objective and subjective refraction more accurately and efficiently than conventional autorefraction or retinoscopy under cycloplegia [27]. Other investigations comparing portable wavefront-based autorefractors with standard methods confirmed that results are generally consistent for spherical equivalent, while astigmatism remains more variable depending on the device and measurement principles [28,29,30]. Taken together, these studies support the notion that our findings with this system align with the broader evidence from similar technologies, reinforcing its potential for screening and refractive assessment in both clinical and community settings.

Joseph et al. compared the accuracy of QSF with that of SCR in providing well-tolerated eyeglasses in 400 participants aged between 18 and 75 years (28.4 ± 6.6 years) and reported a strong correlation between SE (r = 0.97, *p* < 0.001) with a mean difference of −0.07 D [17]. To mitigate the effects of accommodation, all measurements were taken with a +2.00 D fogging lens attached to the device [31]. In our study, the mean differences in the refractive values of the QSF and SCR for SE, even with complete accommodation control by cycloplegia, were −0.83 ± 1.02 D (Table 4).

Hernandez et al. used QSF in 75 adults aged 53 ± 14 years (range: 20 to 74 years) and compared its results with those of SCR conducted by two trained health professionals [13]. The mean ± SD of the differences in SE between the QSF and the SCR1 and SCR2 was ±0.24 ± 1.11 (*p* = 0.56) and −0.02 ± 1.09 D (*p* = 0.88), and that between SR1 and SCR2 was −0.26 ± 0.63 D (*p* = 0.47). The differences in astigmatic components were found to be negligible.

Loayza et al. evaluated the ability of the QSF to detect and measure refractive error relative to the gold standard SCR in a population of 101 children aged between 3 and 17 years (10.2 ± 3.45 years) [32]. The accuracy for detecting myopia, anisometropia, and astigmatism was calculated based on the American Association for Pediatric Ophthalmology and Strabismus 2021 guidelines (hyperopia > +4.00 D, anisometropia > 1.25 D, myopia < −3.00 D (children < 48 months) or <−2.00 D (children ≥ 48 months), and astigmatism > 3.00 D (<48 months) or >1.75 D (≥48 months)) [33]. The area under the receiver operating characteristic curve was greater than 0.75 for all three conditions, indicating that the QSF has good predictive ability for detecting myopia, anisometropia, and astigmatism.

In our study, the accuracy of QSF without and under cycloplegia, for detecting significant refractive errors (hyperopia ≥ +2.00 D, myopia ≤ −0.75 D, astigmatism ≥ 1.00 DC and anisometropia ≥ 1.00 D), was assessed using ROC curve analysis with the SCR as the gold standard. The values of AUC without and under cycloplegia were for hyperopia (0.882 and 0.909), myopia (0.869 and 0.930), astigmatism (0.825 and 0.826), and anisometropia (0.538 and 0.550), respectively. An AUC above 0.9 is excellent, 0.8 to 0.9 is very good, 0.7 to 0.8 is good, 0.6 to 0.7 is average, and below 0.6 is poor [34].

One advantage of QSF is that it is lightweight, easy to use, and easily portable, meaning that the individual identified in the visual screening may also be submitted to refractometry in the same place, and then they can receive a glasses prescription, thus reducing absenteeism, which occurs when the participant is referred for examination in another location [35].

### Limitations

One essential limitation of this study is its small sample size. The measurements and analysis of only the refraction of RE may not fully reflect real-world conditions or bilateral variability. Nevertheless, the accuracy of refraction measures by QSF, both with and under cycloplegia, was assessed using ROC curve analysis with SCR as the gold standard, based on data obtained from both eyes. Another limitation is the fact that QSF has a range (spherical correction from −13.00 D to +10.00 D and cylindrical correction from −8.00 DC to +8.00 DC) that covers most of the population; therefore, there is always the possibility of high refractive errors being left out. Furthermore, no analysis related to the age of the participants was conducted.

## 5. Conclusions

The average bias for the QSF was moderately negative for SC and SE, indicating that, on average, the QSF tended to underestimate these measures relative to SCR. The QSF demonstrated good predictability in detecting significant refractive errors in low-resource settings.

## Figures and Tables

**Table 1:** Right-eye refraction values obtained by QSF without cycloplegia in a sample of 100 adults aged 18 to 65 years. Manacapuru, Amazonas, 2025.

	Variables	Mean ± SD ^1^	Median	Min	Max
QSF *	SC ^2^	−0.66 ± 1.70	−1.00	−3.75	3.75
	CC ^3^	−0.83 ± 0.75	−0.50	−3.75	0.00
	CA ^4^	9.53 ± 54.06	6.00	−89	90
	SE ^5^	−1.08 ± 1.67	−1.19	−4.25	3.38
	MV90 ^6^	0.10 ± 0.51	0.00	−0.99	1.78
	MV135 ^7^	0.04 ± 0.22	0.03	−0.58	0.86

^1^ SD: standard deviation; ^2^ SC: spherical component; ^3^ CC: cylindrical component; ^4^ CA: axis of cylindrical component; ^5^ SE: spherical equivalent; ^6^ MV90: magnitude vector on the 90° axis; ^7^ MV135: the difference between diopter components projected on the 135° axis and the 45° axis; * QSF: refraction values obtained by QSF without cycloplegia and RU. Wilcoxon’s signed-rank test; statistically significant results with a *p*-value less than 0.05.

**Table 2:** Right-eye refraction values obtained by SCR under cycloplegia in a sample of 100 adults aged 18 to 65 years. Manacapuru, Amazonas, 2025.

	Variables	Mean ± SD ^1^	Median	Min	Max
SCR *	SC ^2^	0.42 ± 1.32	0.00	−2.50	2.75
	CC ^3^	−0.71 ± 0.63	−0.50	−3.50	0
	CA ^4^	17.09 ± 48.40	7.50	−85	90
	SE ^5^	0.07 ± 1.38	−0.25	−2.50	2.50
	MV90 ^6^	0.10 ± 0.41	0.00	−0.74	1.75
	MV135 ^7^	0.06 ± 0.21	0.02	−0.47	0.96

^1^ SD: standard deviation; ^2^ SC: spherical component; ^3^ CC: cylindrical component; ^4^ CA: axis of cylindrical component; ^5^ SE: spherical equivalent; ^6^ MV90: magnitude vector on the 90° axis; ^7^ MV135: the difference between diopter components projected on the 135° axis and the 45° axis; * SCR: refraction values obtained by SCR with cycloplegia. Wilcoxon’s signed-rank test; statistically significant results with a *p*-value less than 0.05.

**Table 3:** Univariate analysis of differences between right-eye refraction values obtained without cycloplegia by QSF and SCR in a sample of 100 adults aged 18 to 65 years. Manacapuru, Amazonas, 2025.

	Variables	Mean ± SD ^1^	Median	IC 95%	*p*-Value
QSF-SCR *	SC ^2^	−1.08 ± 1.17	−1.00	−1.37...−1.00	<0.0001 *
	CC ^3^	−0.13 ± 0.63	0.00	−0.25...0.00	0.0365 *
	CA ^4^	−4.96 ± 42.18	−1.00	−8.00...+2.50	0.3532
	SE ^5^	−1.15 ± 1.15	−1.12	−1.31...−0.94	<0.0001
	MV90 ^6^	0.00 ± 0.44	0.00	−0.05...+0.07	0.7271
	MV135 ^7^	−0.02 ± 0.21	−0.03	−0.05...+0.01	0.2896

^1^ SD: standard deviation; ^2^ SC: spherical component; ^3^ CC: cylindrical component; ^4^ CA: axis of cylindrical component; ^5^ SE: spherical equivalent; ^6^ MV90: magnitude vector on the 90° axis; ^7^ MV135: the difference between diopter components projected on the 135° axis and the 45° axis; * QSF-SCR: the difference between refraction values obtained by QSF without cycloplegia and SCR. Wilcoxon’s signed-rank test; statistically significant results with a *p*-value less than 0.05.

**Table 4:** Univariate analysis of differences between right-eye refraction values obtained with cycloplegia by QSF and SCR in a sample of 100 adults aged 18 to 65 years. Manacapuru, Amazonas, 2025.

	Variables	Mean ± SD ^1^	Median	IC 95%	*p*-Value
QSF-SCR *	SC ^2^	−0.81 ± 1.07	−0.75	−1.12...−0.63	<0.0001
	CC ^3^	−0.05 ± 0.61	0.00	−0.13...0.00	0.0850
	CA ^4^	−14.70 ± 60.40	0.00	−17.50...+2.00	0.2896
	SE ^5^	−0.83 ± 1.02	−0.62	−1.00...−0.63	<0.0001 *
	MV90 ^6^	−0.02 ± 0.35	−0.01	−0.06...+0.04	0.6830
	MV135 ^7^	−0.03 ± 0.26	0.00	−0.06...+0.03	0.5037

^1^ SD: standard deviation; ^2^ SC: spherical component; ^3^ CC: cylindrical component; ^4^ CA: axis of cylindrical component; ^5^ SE: spherical equivalent; ^6^ MV90: magnitude vector on the 90° axis; ^7^ MV135: the difference between diopter components projected on the 135° axis and the 45° axis; * QSF-SCR: the difference between refraction values obtained by QSF with cycloplegia and SCR. Wilcoxon’s signed-rank test; statistically significant results with a *p*-value less than 0.05.

## Data Availability

The original contributions presented in this study are included in the article/Supplementary Material. Further inquiries can be directed to the corresponding authors.

## Supplementary Materials

The following supporting information can be downloaded at: https://www.mdpi.com/article/10.3390/vision9040094/s1, Supplementary Figure S1: Sample size calculation based on the standard deviation of the difference between QuickSee and subjective refraction under cycloplegia; Supplementary Figure S2: Bland–Altman plot comparing the spherical equivalent obtained with QuickSee and subjective refraction under cycloplegia.

## Abbreviations

The following abbreviations are used in this manuscript:

QSF	QuickSee Free (portable autorefractor)
SCR	Subjective Clinical Refractometry
SC	Spherical Component
CC	Cylindrical Component
CA	Cylindrical Axis
SE	Spherical Equivalent
MV90	Magnitude Vector on the 90° axis
MV135	Magnitude Vector on the 135° axis
ROC	Receiver Operating Characteristic curve
AUC	Area Under the Curve
VA	Visual Acuity
FDA	Food and Drug Administration
DCs	Cylindrical Diopters
D	Diopter
RE	Right Eye
AAPOS	American Association for Pediatric Ophthalmology and Strabismus
VI	Visual Impairment
UREs	Uncorrected Refractive Errors
