# Supplementary material for: Clinical Evaluation of an Affordable Handheld Wavefront Autorefractor in an Adult Population in a Low-Resource Setting in the Amazonas
Source: Vision (Basel). 2025 Nov 6;9(4):94. doi: 10.3390/vision9040094 (PMC12641641; doi:10.3390/vision9040094)
Supplement: Supplementary file 1 [file vision-09-00094-s001.zip › vision-3842192-supplementary.pdf]

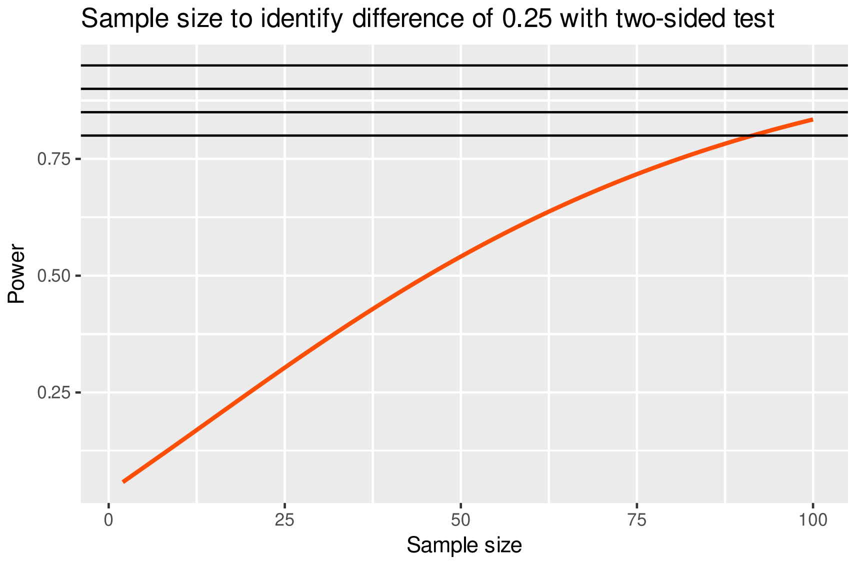

Supplementary Figure S1. Sample size calculation based on the standard deviation of the difference between QuickSee and subjective refraction under cycloplegia. The analysis indicated that 92 individuals would be sufficient to detect a difference of 0.25 diopters with 80% power and a significance level of 0.05.

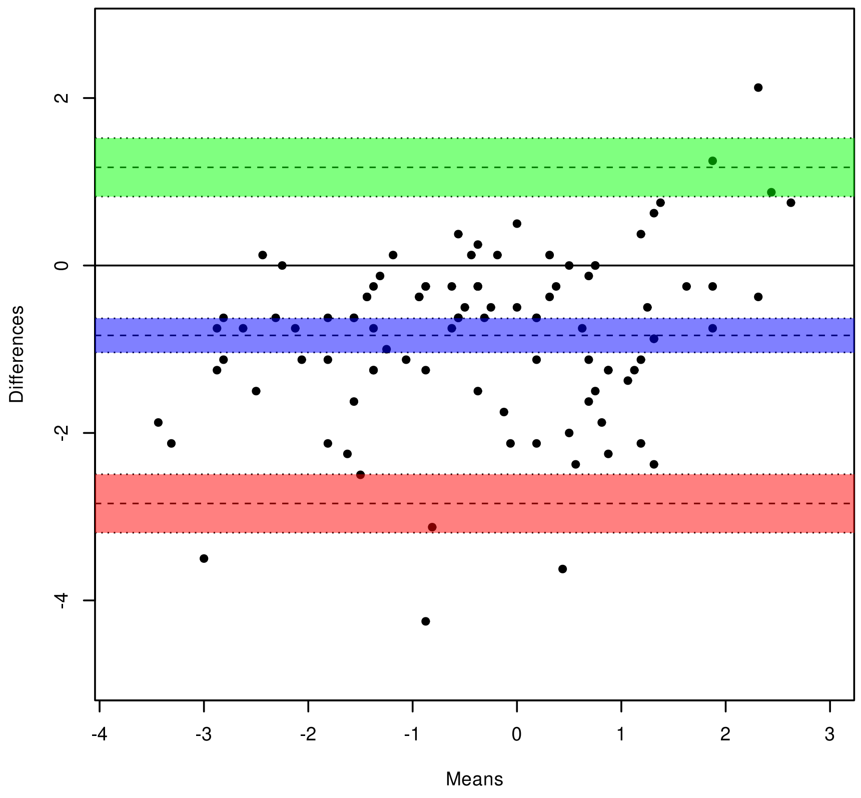

Supplementary Figure S2. Bland–Altman plot comparing the spherical equivalent obtained with QuickSee and subjective refraction under cycloplegia. The analysis shows a mean bias of –0.835 D, with 95% confidence interval from –1.0382 to –0.6318, indicating a systematic underestimation of the spherical equivalent by QuickSee.
